# Dual careers as sustainable careers for performing artists in times of crisis. A contextual approach to the construct of a sustainable arts career

**DOI:** 10.1371/journal.pone.0314933

**Published:** 2024-12-05

**Authors:** Patrycja Mizera-Pęczek, Anna Krasnova, Magdalena Sasin, Aleksandra Sieczych-Kukawska

**Affiliations:** 1 Department of Human Resources Management, Faculty of Management, University of Lodz, Lodz, Poland; 2 Department of Art Education and Pedagogy of Creativity, Faculty of Educational Sciences, University of Lodz, Lodz, Poland; Universidad Santiago de Cali, COLOMBIA

## Abstract

For an individual, a sustainable career is a sequence of events in one’s professional life that both brings professional satisfaction to the career actor, but also allows them to stay healthy, fit, satisfied with their non-work life, feel stable and secure, and free to decide their professional future. It is challenging to pursue a career as a performing artist in such a way that the artist can say of their career that it is sustainable. Indeed, research shows that artists struggle with job instability, being paid below expectations, high competition for lucrative career opportunities and the loss of psycho-physical fitness, which is important in the profession. Dual career is one of the paths for artists to build a conscious sustainable career. Inspired by research on dual careers for athletes, we found many similarities between the sport and artistic settings, mainly related to the short-term nature of these professions. The idea of an artist pursuing a dual career in such a way that they start preparing for an alternative career path already during their artistic training seems to be a potentially attractive career management strategy to facilitate career reorientation. This study investigates the opinions of performing artists regarding the opportunities to pursue a sustainable career through conscious preparation for career reorientation. A total of 111 performers from Poland participated in the survey. Assessments of the situation of artists in the labour market during the pandemic crisis, their attitudes towards the end/descent of their artistic careers, their opinions on alternatives to practising as a performing artist, and their assessments of the potential for pursuing a dual career strategy were investigated. The survey revealed that the opinions of artists are diverse and ambiguous, probably due to the internal diversity of this professional community. Importantly, a significant group of respondents in our survey said that it was the pandemic that made them aware of the instability of a career as a performing artist (56.7%). The analysis of the research results shows what career self-management strategies the respondents use. Remarkably, for a group of respondents, the pandemic crisis has become a determinant for taking steps towards sustainable career management.

## Introduction

Professional reorientation of artists entails artists revising their previous plans to pursue an artistic career (in the occupational/professional sense) [1]. Consequently, reorientation may lead to a change of employer, job position, scope of duties or even to a change of profession and industry in which the reoriented person would like to continue their career [2, 3]. In the turbulently changing economic climate of the VUCA world (Volatile, Uncertain, Complex, Ambiguous) [4], career reorientation may be an attribute of any career pattern [3, 5] but it is most extensively described in the literature in terms of short-term occupations, which may include that of an athlete or dancer [6–8]. In contrast, there is a cognitive gap in terms of the nature of artists’ career reorientation as a result, either expected or not, of the end of their artistic career [9].

Professional reorientation is a process that is difficult to evaluate. It appears to be a positive coping strategy for an artist in a career crisis, when the artist is required to adapt to the new realities of the labour market, but it can be perceived as a negative phenomenon by artists themselves when it unequivocally leads to a career winding down against the artist’s will. Career reorientation should therefore be acknowledged not only from an economic perspective and examined not only in terms of the benefits it may provide to the reoriented person, but above all in terms of the subjective lived experiences of the artists themselves [10].

The pandemic and the subsequent destabilisation of the economic situation of cultural institutions [11] have contributed to a resurgence of research interest in the issue of professional reorientation in culture, particularly in the performing arts [12–14]. Previous publications on the issue of career reorientation mostly address the professional situation of dancers (e.g. ballet) who end their artistic careers due to physical and health limitations [15]. Any sudden restriction to continue a career is referred to as a career crisis [16]. Previous research has established that artistic career crises are primarily influenced by personal circumstances, such as loss of health and fitness or the desire for stability resulting from starting a family [6, 17]. However, the pandemic exacerbated artistic career crises at the subject level (when conditioned by changes in the setting of arts organisations), as artists were unable to do their work at the expected level and scale due to the closure of many cultural institutions [11, 18]. Notably, the weakening of professional commitment also occurred in other professions [19].

It appears that the pursuit of dual careers by artists is a tool to curb the negative effects of forced professional reorientation. It could even be argued that it is a kind of training to prepare oneself for a smooth transition between professions or even the nature of work. It is also a vehicle for building employability [20], not least by building a sense of self-efficacy [21]. Therefore, one could argue that it is one of the options for building a sustainable career [5, 9].

The need for increased intellectual development and physical and emotional commitment to perfecting one’s craft as an actor, singer or dancer may limit the unfolding of an alternative career path (dual career), but it should be presumed that in an era of widespread access to hybrid and flexible educational institutions, this is not infeasible.

The downsizing of cultural institutions in 2020 is an unprecedented situation; its long-term consequences are difficult to clearly predict at present [22]. However, we are interested in determining whether the pandemic has contributed to an increased awareness of the unpredictability of artistic careers among stakeholders themselves [18, 23]. Although we are now seeing the effects of the unfreezing of cultural institutions, it still seems interesting whether the ‘hot’ events of 2020 have contributed to a shift in thinking about careers in a way that makes them more sustainable, including stable, secure, predictable and providing for the broader well-being of the career actor [24]. Hence, the objective of this paper is to identify the views of performers on sustainable career construction. We have hypothesised that sustainable artistic careers are those when an artist can balance artistic career objectives with non-artistic or peri-artistic professional objectives, e.g. by pursuing a dual career strategy.

Importantly, literature studies have shown that researchers focus their attention on different groups of artists. This manuscript focuses on performing artists (actors, dancers, vocalists and instrumentalists) whose specific profession is presence on stage and contact with the audience "here and now".

## Theoretical background

### Career duality as a strategy for managing artistic careers

The study of cultural institutions is an underdeveloped sub-discipline of science in Poland. The discourse lacks the results of long-term, longitudinal and comparative research [25]. However, the careers of professional performing artists in Poland may be described as unstable, uncertain, requiring dedication and sacrifice. Similarly, Wyszomirski and Chang [26] comment on the careers of professional artists, in a global context:

Artists are an enigma: they challenge many of the accepted notions of what it means to be members of a professional group in this society. On the one hand, they tend to undertake an extended period of formal training, maintain affiliations with professional organizations, and develop a strong sense of identification with their chosen field. On the other hand, they are subject to a number of highly contrasting trends. Their rates of unemployment and underemployment are marked, their income and the status they are accorded is low, and the degree of control and predictability they have over career lines is low relative to other professional groups.

Published reports on the professional situation of artists in Poland confirm the similarity of artistic career trajectories in the international background [27–29]. An artistic career pursued in Poland, especially in the area of performing arts, is associated with an early start of formal artistic education (in the case of music schools, parallel education is provided in general schools and music schools—afternoons, or education in general schools with a music profile. In the case of training for dancers, In several largest cities in Poland, general ballet schools are run, attended by young people aged 10 to 19. At the age of 19, provided they pass the high school leaving examination, it is possible to pursue higher education at: theater academies and music academies.

In analysing contemporary discourse on careers, it is important to assert that careers have a personal character [30, 31]. Successful career development requires increased career self-management, and contemporary career orientations appropriately emphasise the importance of self-direction, guided by individuals’ values in career management and flexibility in professional decision-making [32, 33]. Career self-management seeks to make careers more sustainable [34, 35]. This personal approach to career management is the first pillar of career sustainability. The next are the background, i.e. the conditions under which careers are structured [36] and the time perspective over which career sequences take place [35]. A sustainable career requires the creative actions of both employees and their employers, who are often responsible for the career background. Sustainable careers therefore require more than individual agency, such as building sustainable career resources (skills, knowledge, interpersonal competences) [37] and actions, too, to enable the dynamic use of these resources in specific circumstances [36].

On this ground, the notion of dual careers, involving the skilful combination of different career paths or professional and educational activities, has emerged [38, 39]. Morris et al [40] underline the richness of the organisational experience of dual career for athletes, which is crucial for them to cope with adversity, protect themselves from poor mental health or burnout, and retain a perspective for them. In addition, athletes who undertake a dual career are often better prepared for sporting retirement and experience a more successful transition out of sport compared to retired athletes who followed a sport-only path. However, to take note, Quinaud et al. [41] remark that the skilful blending of careers in, for example, academia and sport is determined not only by the intention to manage an artistic career in a more balanced way, but also by the sporting level represented, the assessment of the prospects of a sport career, also the self-awareness and personal values of the athletes, and the institutional support of the dual career from sport and academia. Cultural background may also be relevant in this respect. Nevertheless, the pursuit of dual careers is valued for qualities such as preventing the risk of sport or academic burnout and dropping out [41] and to cope with unpredictable situations such as the emergency of the COVID-19 pandemic [42].

In view of the potential benefits of dual careers [8], but also the similarity of sport and artistic careers in terms of long-term and exhaustive professional preparation [17], it appears reasonable to discern both the personal and environmental conditions that would facilitate dual careers precisely in creative environments. The highly individual nature of artistic careers and the specificity of the creative process are not factors that protect artists from career crises [43].

Many artists begin their careers at a very young age, at that time combining the roles of, for example, singer, dancer, actor and student with varying degrees of success. It is difficult to predict whether an artist’s early career will evolve to a level that will provide the artist with professional stability and a decent salary [44]. Similarly, when high school graduates choose to study performing arts, they are uncertain about a stable career in this labour market, which is challenging for workers [35]. A study of the conditions faced by dancer-artists in Poland shows that a significant proportion of workers lack stable employment conditions and social security. The consequences of this situation also include problems related to health, family life, and long-term career planning [45–47]. Hopper et al. even go on to write that “dancers dedicate their lives to their art [17]”. Career reorientation and retraining programmes are not yet widely available to all stakeholders [1], and good practices for implementing dual careers are not well established in organisations.

Similarly, in the case of the study of musicians’ careers, researchers report that music careers are demanding, with physical burdens, performance anxiety and mental health problems that can result from the strain of the creative process [48].

Therefore, in parallel with a demanding artistic education, artists should foster other competences [49] that will allow them, at the end of their career, or if they wish to retrain as a result of career dissatisfaction, to smoothly return to a non-artistic or peri-artistic profession [50] or simply, through a sense of security and competence, to draw more professional satisfaction from their artistic career [28].

This would help overcome job insecurity, that is, employees’ fears of threats to future job continuity [2, 48, 49]. Uncertainty is a subjective experience, and reflects an expectation of a fundamental and involuntary change in career prospects. In terms of performers, this uncertainty is amplified by a number of factors, such as those related to age and transient physical attractiveness (thus physical fit for the roles created) or physical fitness. Research shows that job insecurity has consequences for a range of employee problems, including physical health, mental health, job satisfaction, productivity and ultimately the intention to find other employment. While the effects of job loss are immediate, feelings of instability are associated with prolonged uncertainty about future employment prospects, and such uncertainty may have far-reaching consequences [27].

In tune with the holistic approach to career management, which in today’s world of work is to allow for parallel academic, professional, psychological, psychosocial and financial growth over the course of an individual’s life [33], a dual career involving the diversification of professional activities [51] is a strategy worth considering for artists as well.

## Current study

### Data and methods

The presented theoretical rationale led us to identify performing artists’ attitudes towards pursuing dual careers, in light of the unstable job market conditions for artists in Poland. Previous literature studies on the professional situation of artists have shown that artists’ career reorientation is one of the important tools for career management in a personal, organisational or social crisis. Learning about the opinions of artists themselves about solutions to support career diversification will help to fill the existing research gap.

In this project, we formulated the research problem as follows: What are the constraints on the pursuit of sustainable careers by performing artists. The research problem is detailed in the following research questions:

Q.1. What is the assessment of the situation of artists in the job market amidst the pandemic crisis?
Q.2. What are the attitudes of artists towards the end/descent of their artistic careers?
Q.3. What are the alternatives to practising as a performing artist in the respondents’ evaluations?
Q.4. What are the potential options for pursuing a dual career strategy in the views of the respondents?

We decided to include gender, seniority (total and artistic) and pursuing a single or dual career in the analysis of respondents’ statements.

We conducted an exploratory and explanatory project to answer the research questions. We carried out the quantitative research using a survey method. An external contractor, a research company based in Warsaw, was responsible for carrying out the survey. Survey forms (S1 File) were distributed to performing artists: actors, dancers, and musicians, in order to collect facts about their professional experiences. The objective of the research was to learn the artists’ opinions about potential opportunities to pursue dual careers in the context of the pandemic crisis. The selection of the sample was purposive. The criteria for selection for the survey were a performing artist’s livelihood from work of an artistic nature and willingness to participate in the survey. We obtained the research results from 111 completed surveys, which, with a population size of approximately 27,000 performers in Poland [45], allows us to present reliable results for the Polish performing arts labour market (for a fraction size of 0.5 and a confidence level of 95%, the maximum error is 9%). The research was conducted with full attention to ethics clearance. Participants of the research were informed about the purpose of the research and its anonymous character.

In order to answer the research question, statistical analyses were performed using IBM SPSS Statistics 23 (S1 Dataset). Basic descriptive statistics, a series of non-parametric tests of difference for two independent samples Mann-Whitney U and Spearman’s rho correlation analyses were performed. The level of statistical significance was set at α < 0.05.

The findings on the characteristics of the collected research sample and basic descriptive statistics are presented below.

### Research sample characteristics

A total of 111 people took part in the survey; 61 women, 47 men and two non-binary people, one person declined to specify their gender in the survey. Due to the small number of non-binary people, only women and men were included in further analyses related to the comparison of responses by gender (N = 108). The remaining analyses were performed on all observations.

### Age

Of the research sample obtained, six people were under 20 years of age (5.40%), 49 people were between 21 and 39 years of age (44.14%), 43 people were between 31 and 40 years of age (38.74%), 7 people were between 41 and 50 years of age (6.31%), 4 people were between 51 and 60 years of age (3.60%), and 2 people were over 60 years of age (1.80%).

### Education

The respondents were asked about both their general education and their educational background in the field (arts). Among the respondents, the majority had tertiary education (70 people, 63.06% of the total sample surveyed), 36 people had secondary education (32.43%), two people had lower secondary education (2%), another two had basic vocational education (0,18%) and one person had primary education (0.90%). Almost half of the respondents (48.65%) have no artistic education, 23 people declared a completed university degree in an artistic field (20.72%) and an equal group of respondents declared a completed secondary arts school (20.72%); 11 people have a completed primary school with an artistic profile (9.90%).

### Job tenure

The survey measured both general seniority and artistic seniority. Among the respondents, 35.14% identified the tenure of their total work experience in the range of 6–10 years, 23.42% in the range of 1–5 years, while 20.72% of the respondents have been working for 11–15 years, 15.32% of the respondents have been working for 16–17 years, four people have been working for more than 20 years (3.60%), and two people have been working for less than one year (1.80%).

Among the respondents, 35.14% defined the tenure of the artistic profession in the range of 1–5 years; 35 people have been working in the artistic profession in the range of 6–10 years (31.53%), 23 people have been working for 11–15 years (20.72%), two people have been working for more than 20 years (1.80%), while three people have been working for less than one year (2.70%).

## Results

Respondents expressed their opinions on a 5-point response scale. The method of coding was as follows: I strongly agree with the statement was expressed on the scale as a value of 5 and I agree with the statement as 4, I disagree with the statement as 2 and I strongly disagree with the statement as 1. A value of 3 was ‘hard to say’. Therefore, a 5-point Likert scale was used. The survey included four constructs consisting of five items each. The first construct concerned the assessment of the job market situation in the pandemic crisis. An example item in this construct was, e.g. “It was the pandemic that made me realise that a career as a performing artist is unstable”. Another construct concerned artists’ attitudes towards ending their artistic careers. We asked, e.g. whether ending an artistic career is a failure in an artist’s professional life. We also explored opinions about alternatives to the performing arts profession by asking, e.g. whether formal support for retraining artists is implemented sufficiently. We also learnt about assessments of the potential of pursuing a dual career (e.g. “A dual career is the expected way of pursuing a career in my professional environment”). The reliability of the measures in this study was assessed by two commonly used measures: Cronbach’s Alpha and Composite Reliability (CR). Using both reliability criteria, we see that all constructs meet typical requirements–values greater than 0.7 are suggested. In a first step, descriptive statistics were checked for all analysed items (Table 1).

**Table 1:** Descriptive statistics for the analysed variables.

	N	Minimum	Maximum	Mean		Standard deviation	Variation	Skewness		Kurtosis	
	Statistics	Statistics	Statistics	Statistics	Standard error	Statistics		Statistics	Standard error	Statistics	Standard error
**Artists’ view of the job market situation in the pandemic crisis**											
It was the pandemic that made me realise that a career as a performing artist is unstable.	111	1	5	**3,45**	**,114**	1,204	1,450	-,422	,229	-,860	,455
It was the pandemic that made me realise that a career as a performing artist did not provide me with a decent standard of living.	111	1	5	**3,01**	**,126**	1,325	1,754	-,112	,229	-1,206	,455
It was the pandemic that made me realise that I should plan an alternative career path.	111	1	5	**3,17**	**,124**	1,306	1,707	-,274	,229	-1,070	,455
It was the pandemic that prompted me to pursue an education preparing me for a profession other than the performing arts profession.	111	1	5	**2,64**	**,133**	1,400	1,960	,163	,229	-1,350	,455
It was the pandemic that prompted me to seek additional work in a profession other than the performing arts profession.	111	1	5	**2,93**	**,141**	1,481	2,195	-,011	,229	-1,470	,455
**Artists’ attitudes to career end/descent**											
Ending an artistic career is a setback in an artist’s professional life. [inverted scale]	111	1	5	**2,97**	**,130**	1,372	1,881	-,037	,229	-1,225	,455
I will be able to give up my artistic career if it negatively affects my physical health.	111	1	5	**3,18**	**,109**	1,154	1,331	-,107	,229	-,990	,455
I will be able to give up my artistic career if it negatively affects my mental health (e.g. experiencing negative emotions, excessive workload, feeling stressed and pressured)	111	1	5	**3,27**	**,114**	1,198	1,435	-,313	,229	-,832	,455
I will be able to give up my artistic career if it interferes with my private life (e.g. family, social life).	111	1	5	**2,79**	**,110**	1,161	1,348	,095	,229	-,821	,455
I will be able to give up my artistic career if it does not provide me with a decent standard of living.	111	1	5	**2,97**	**,113**	1,187	1,408	-,013	,229	-,944	,455
**Alternatives to practising as a performing artist**											
Pedagogical education makes it easier for artists to find a rewarding job after their artistic career is over.	111	1	5	**3,19**	**,110**	1,156	1,337	-,451	,229	-,460	,455
Non-artistic (other than pedagogical) education makes it easier for artists to find a rewarding job after their artistic career is over.	111	1	5	**3,77**	**,094**	,988	,976	-,683	,229	-,021	,455
Having only an art degree is sufficient for an artist to find a rewarding job after their artistic career is over.	111	1	5	**2,62**	**,097**	1,019	1,037	,189	,229	-,601	,455
Formal support to artists for vocational retraining is properly implemented (e.g. through existing vocational retraining programmes).	111	1	5	**2,53**	**,090**	,952	,906	,037	,229	-,347	,455
Cultural institutions where performing artists are employed properly prepare artists for the need for professional reorientation (retraining).	111	1	5	**2,39**	**,094**	,992	,985	,347	,229	-,430	,455
**Potential opportunities for a dual career strategy**											
A dual career (combining artistic and non-artistic work) is the expected way to pursue a career in my professional environment.	111	1	5	**3,05**	**,108**	1,139	1,298	-,277	,229	-,797	,455
During my art education, I was prepared to have to change careers.	111	1	5	**2,27**	**,104**	1,095	1,199	,373	,229	-,837	,455
During my art education, I was encouraged to improve my non-artistic competences.	111	1	5	**2,87**	**,115**	1,207	1,457	-,164	,229	-1,134	,455
A dual career hinders the artistic progress of the professional performing artist. [inverted scale]	111	1	5	**2,71**	**,116**	1,224	1,498	,117	,229	-1,091	,455
During art education, one can prepare for other professions at the same time without much trouble.	111	1	5	**3,02**	**,106**	1,112	1,236	-,198	,229	-,709	,455

Source: own research.

The analysed Table 1 presents descriptive statistics regarding artists’ attitudes toward the job market situation during the pandemic and their career plans. A significant finding is that artists recognise the instability of their careers in the context of the pandemic crisis, with a mean score of 3.45 (on a scale from 1 to 5) indicating that the pandemic has highlighted this instability. The mean score of 3.01 reflects a neutral perception regarding the idea that an artistic career does not provide a decent standard of living, while a mean of 3.17 shows moderate acknowledgment of the need to plan alternative career paths. In terms of seeking additional work and education, the means of 2.64 and 2.93 suggest a lower interest in these options. Regarding attitudes toward the end of an artistic career, a mean of 2.97 indicates a near-neutral view on whether ending a career is seen as a setback. There is a greater readiness to abandon the artistic career if it negatively impacts physical and mental health, reflected in the means of 3.18 and 3.27, respectively. A mean of 2.79 shows moderate concern about how an artistic career affects personal life. Another key conclusion concerns alternatives to practicing as an artist. A mean of 3.19 suggests that artists perceive pedagogical education as beneficial for future job opportunities, while a mean of 3.77 strongly supports the idea that nonartistic education aids in finding employment. On the contrary, a mean of 2.62 indicates concerns about the value of an art degree in the job market. In terms of support for vocational retraining, a mean of 2.53 indicates that artists generally view institutional support as insufficient, while a mean of 2.39 with respect to the preparation of cultural institutions for professional reorientation confirms these concerns. Lastly, regarding the potential for dual career strategies, a mean of 3.05 reflects moderate expectations about combining artistic and non-artistic work, while a mean of 2.27 suggests a low evaluation of how well art education prepares individuals for career changes. Finally, a mean of 2.71 indicates mixed feelings about the impact of dual careers on artistic progress.

In a further step of the statistical analyses, we examined the research questions. In order to answer the questions, we verified the difference in the degree of agreement with individual statements according to the gender of the respondents (female vs. male) and differences according to the career path followed (dual vs. single). We also checked the correlations of the individual statements with professional seniority (total and seniority as an artist). In the manuscript, we presented only the results of studies that were statistically significant.

### Assessment of the situation of artists on the job market in the pandemic crisis

In order to see what the assessment of the artists’ job market position in the pandemic crisis is, we checked whether gender matters in the assessment of the artists’ job market situation. The detailed results of the comparison of statements regarding the assessment of the job market position in the pandemic crisis by gender are presented in Table 2. Within the analyses, no differences were observed between men and women in the assessment of the job market position of artists in times of crisis. Interestingly, no differences were also observed between those pursuing a dual career and those focused on a single career as an artist. The detailed results are presented in Table 3.

**Table 2:** Degree of agreement with individual statements regarding the assessment of the position of artists in the pandemic crisis, compared by gender of respondents (N = 108).

	Woman (n = 61)		Man (n = 47)			
Dependent variable	*Mdn*	*M*_rank_	*Mdn*	*M*_rank_	Mann-Whitney *U*	
It was the pandemic that made me realise that a career as a performing artist is unstable.	4	52,77	4	56,74	*U* = 1328,00; *p* = 0,497	
It was the pandemic that made me realise that a career as a performing artist did not provide me with a decent standard of living.	3	54,76	3	54,16	*U* = 1417,50; *p* = 0,919	
It was the pandemic that made me realise that I should plan an alternative career path.	3	52,34	4	57,31	*U* = 1301,50; *p* = 0,401	
It was the pandemic that prompted me to pursue an education preparing me for a profession other than the performing arts profession.	3	54,35	3	54,69	*U* = 1424,50; *p* = 0,954	
It was the pandemic that prompted me to seek additional work in a profession other than the performing arts profession.	3	54,22	4	54,86	*U* = 1416,50; *p* = 0,914	

Source: own research.

**Table 3:** Degree of agreement with individual statements on the assessment of the position of artists in the pandemic crisis, compared by career path pursued (N = 111).

	Dual (n = 72)		Single (n = 39)		
Dependent variable	*Mdn*	*M*_rank_	*Mdn*	*M*_rank_	Mann-Whitney *U*
It was the pandemic that made me realise that a career as a performing artist is unstable.	4	55,19	4	57,49	*U* = 1346,00; *p* = 0,710
It was the pandemic that made me realise that a career as a performing artist did not provide me with a decent standard of living.	3	58,65	3	51,10	*U* = 1213,00; *p* = 0,226
It was the pandemic that made me realise that I should plan an alternative career path.	4	58,31	3	51,74	*U* = 1238,00; *p* = 0,292
It was the pandemic that prompted me to pursue an education preparing me for a profession other than the performing arts profession.	3	56,49	2	55,09	*U* = 1368,50; *p* = 0,821
It was the pandemic that prompted me to seek additional work in a profession other than the performing arts profession.	3	57,42	2	53,38	*U* = 1302,00; *p* = 0,518

Source: own research.

In the next step, it was investigated whether there was a correlation between agreement with the individual statements regarding artists’ assessment of the job market situation and job tenure (Table 4). No statistically significant relationships were observed.

**Table 4:** Correlations of survey items on the assessment of the job situation of artists in the pandemic crisis and job tenure.

		Job tenure (total)	Job tenure in the arts
It was the pandemic that made me realise that a career as a performing artist is unstable.	Spearman’s *rho*	0,09	0,02
	relevance	0,362	0,859
It was the pandemic that made me realise that a career as a performing artist did not provide me with a decent standard of living.	Spearman’s *rho*	0,12	0,04
	relevance	0,224	0,656
It was the pandemic that made me realise that I should plan an alternative career path.	Spearman’s *rho*	-0,05	-0,07
	relevance	0,635	0,479
It was the pandemic that prompted me to pursue an education preparing me for a profession other than the performing arts profession.	Spearman’s *rho*	0,00	-0,01
	relevance	0,994	0,906
It was the pandemic that prompted me to seek additional work in a profession other than the performing arts profession.	Spearman’s *rho*	-0,03	0,00
	relevance	0,777	0,964

Source: own research.

### Attitudes of artists towards the end/descent of their artistic career

In the next step, differences according to the gender of the respondents (Table 5) and the career path pursued (Table 6) in the degree of agreement with statements representing artists’ attitudes towards the end of their artistic careers were investigated. Within the analyses, a stronger degree of agreement was observed with the statement “I will be able to give up my artistic career if it negatively affects my physical health.” among men (Mdn = 4), U = 1045.50; p = 0.013. Men were also more likely to declare that “I will be able to give up my artistic career if it does not provide me with a decent standard of living.” (Mdn = 3). Interestingly, no significant differences were observed by the career path pursued.

**Table 5:** Degree of agreement with individual statements regarding approaches to ending an artistic career, compared by gender of respondents (N = 108).

	Woman (n = 61)		Man (n = 47)		
Dependent variable	*Mdn*	*M*_rank_	*Mdn*	*M*_rank_	Mann-Whitney *U*
Ending an artistic career is a setback in an artist’s professional life *(inverted scale)*.	3	52,31	3	57,34	*U* = 1300,00; *p* = 0,398
**I will be able to give up my artistic career if it negatively affects my physical health.**	**3**	**48,14**	**4**	**62,76**	***U* = 1045,50; *p* = 0,013**
I will be able to give up my artistic career if it negatively affects my mental health.	3	50,56	4	59,62	*U* = 1193,00; *p* = 0,124
I will be able to give up my artistic career if it interferes with my private life.	3	50,57	3	59,61	*U* = 1193,50; *p* = 0,126
**I will be able to give up my artistic career if it does not provide me with a decent standard of living.**	**3**	**47,82**	**3**	**63,17**	***U* = 1026,00; *p* = 0,009**

Source: own research.

**Table 6:** Degree of agreement with individual statements regarding approach to the end of an artistic career, compared by career path pursued (N = 111).

	Dual (n = 72)		Single (n = 39)		
Dependent variable	*Mdn*	*M*_rank_	*Mdn*	*M*_rank_	Mann-Whitney *U*
Ending an artistic career is a setback in an artist’s professional life *(inverted scale)*.	3	53,53	3	60,56	*U* = 1226,00; *p* = 0,261
I will be able to give up my artistic career if it negatively affects my physical health.	3	52,79	4	61,92	*U* = 1173,00; *p* = 0,140
I will be able to give up my artistic career if it negatively affects my mental health.	3	58,46	3	51,46	*U* = 1227,00; *p* = 0,259
I will be able to give up my artistic career if it interferes with my private life.	3	58,54	3	51,31	*U* = 1221,00; *p* = 0,244
I will be able to give up my artistic career if it does not provide me with a decent standard of living.	3	54,34	3	59,06	*U* = 1284,00; *p* = 0,447

Source: own research.

In the next step, a Spearman’s rho correlation analysis was conducted between the individual statements and job tenure (Table 7). Within the analyses, only a positive correlation of weak strength was observed between total job tenure and the statement “I will be able to give up my artistic career if it interferes with my private life (e.g. family, social life).”, *rho* = 0.19; *p* = 0.044.

**Table 7:** Correlations of survey items reflecting artists’ attitudes towards career end/descent and job tenure.

		Job tenure (total)	Job tenure in the arts
Ending an artistic career is a setback in an artist’s professional life *(inverted scale)*.	Spearman’s *rho*	0,06	0,05
	relevance	0,510	0,628
I will be able to give up my artistic career if it negatively affects my physical health.	Spearman’s *rho*	0,15	0,09
	relevance	0,106	0,359
I will be able to give up my artistic career if it negatively affects my mental health (e.g. experiencing negative emotions, excessive workload, feeling stressed and pressured)	Spearman’s *rho*	0,08	-0,01
	relevance	0,403	0,878
I will be able to give up my artistic career if it interferes with my private life (e.g. family, social life).	Spearman’s *rho*	0,191*	0,12
	relevance	**0,044**	0,217
I will be able to give up my artistic career if it does not provide me with a decent standard of living.	Spearman’s *rho*	0,16	0,00
	relevance	0,101	0,974

Source: own research.

### Alternatives to the performing arts profession as assessed by respondents

In order to investigate respondents’ opinions on alternatives to practising as a performing arts artist, it was examined how much respondents agreed with each statement and whether there were differences in respondents’ evaluations by gender (Table 8) and by career path pursued (Table 9). As part of the analyses, it was observed that males rated artists’ retraining support better (*Mdn* = 3), *U* = 1100.00; *p* = 0.029. Differences were also observed in respondents’ ratings according to the career pursued. Those pursuing dual careers (*Mdn* = 3) were more likely to agree with the statement indicating that “Pedagogical education makes it easier for artists to find a rewarding job after their artistic career is over” than those pursuing single careers, *U* = 1066.50; *p* = 0.030.

**Table 8:** Degree of agreement with statements regarding alternatives to practising as an artist, compared by gender of respondents (N = 108).

	Woman (n = 61)		Man (n = 47)		
Dependent variable	*Mdn*	*M*_rank_	*Mdn*	*M*_rank_	Mann-Whitney *U*
Pedagogical education makes it easier for artists to find a rewarding job after their artistic career is over.	3	58,83	3	48,88	*U* = 1169,50; *p* = 0,089
Non-artistic (other than pedagogical) education makes it easier for artists to find a rewarding job after their artistic career is over.	4	50,77	4	59,34	*U* = 1206,50; *p* = 0,136
**Having only an art degree is sufficient for an artist to find a rewarding job after their artistic career is over.**	**2**	**47,32**	**3**	**63,82**	***U* = 995,50; *p* = 0,005**
**Formal support to artists for vocational retraining is properly implemented.**	**2**	**49,03**	**3**	**61,60**	***U* = 1100,00; *p* = 0,029**
Cultural institutions where performing artists are employed properly prepare artists for the need for professional reorientation.	2	50,31	2	59,94	*U* = 1178,00; *p* = 0,098

Source: own research.

**Table 9:** Degree of agreement with statements regarding alternatives to practising as an artist, compared by career pursued (N = 111).

	Dual (n = 72)		Single (n = 39)		
Dependent variable	*Mdn*	*M*_rank_	*Mdn*	*M*_rank_	Mann-Whitney *U*
**Pedagogical education makes it easier for artists to find a rewarding job after their artistic career is over.**	**3**	**60,69**	**3**	**47,35**	***U* = 1066,50; *p* = 0,030**
Non-artistic (other than pedagogical) education makes it easier for artists to find a rewarding job after their artistic career is over.	4	60,15	4	48,35	*U* = 1105,50; *p* = 0,050
**Having only an art degree is sufficient for an artist to find a rewarding job after their artistic career is over.**	**2**	**50,65**	**3**	**65,88**	***U* = 1018,50; *p* = 0,013**
Formal support to artists for vocational retraining is properly implemented.	3	56,62	3	54,86	*U* = 1359,50; *p* = 0,771
Cultural institutions where performing artists are employed properly prepare artists for the need for professional reorientation.	2	56,42	2	55,22	*U* = 1373,50; p = 0,844

Source: own research.

Very interesting results were obtained for the statement “Having only an art degree is sufficient for an artist to find a rewarding job after their artistic career is over”. Men (*Mdn* = 3), *U* = 995.50; *p* = 0.005, and artists pursuing a single career (*Mdn* = 3), *U* = 1018.50; *p* = 0.013, agree more with the quoted statement. The quoted statement correlates positively and with weak strength with total job tenure (Table 10), *rho* = 0.22; *p* = 0.022.

**Table 10:** Correlations of items covering the issue of alternatives to practising as an artist and job tenure.

		Job tenure (total)	Job tenure in the arts
Pedagogical education makes it easier for artists to find a rewarding job after their artistic career is over.	Spearman’s *rho*	-0,02	-0,06
	relevance	0,798	0,561
Non-artistic (other than pedagogical) education makes it easier for artists to find a rewarding job after their artistic career is over.	Spearman’s *rho*	0,05	-0,03
	relevance	0,637	0,731
Having only an art degree is sufficient for an artist to find a rewarding job after their artistic career is over.	Spearman’s *rho*	,217*	-0,05
	relevance	**0,022**	0,592
Formal support to artists for vocational retraining is properly implemented (e.g. through existing vocational retraining programmes).	Spearman’s *rho*	0,18	0,06
	relevance	0,061	0,553
Cultural institutions where performing artists are employed properly prepare artists for the need for professional reorientation (retraining).	Spearman’s *rho*	0,10	0,01
	relevance	0,290	0,951

Source: own research.

### Potential opportunities for dual career strategies according to respondents

The next step of the analyses investigated the respondents’ assessments of the individual statements describing the potential opportunities for pursuing a dual career strategy, for which purpose it was again investigated whether there were differences in the degree of respondents’ agreement with the individual statements according to gender (Table 11) and the career path pursued (Table 12). No gender differences were observed within the analyses. However, a difference was found in the evaluation of dual careers as an expected way to pursue a career in a professional environment; those pursuing dual careers were significantly more likely to agree with the cited statement (*Mdn* = 3), *U* = 1047.50; *p* = 0.023. Please note also that those pursuing dual careers were also significantly more likely (*Mdn* = 3) to agree with the statement “During art education, one can prepare for other professions at the same time without much trouble.”, *U* = 1087.50; *p* = 0.043.

**Table 11:** Degree of agreement with statements regarding the possibility of pursuing a dual career strategy, compared by gender of respondents (N = 108).

	Woman (n = 61)		Man (n = 47)		
Dependent variable	*Mdn*	*M*_rank_	*Mdn*	*M*_rank_	Mann-Whitney *U*
A dual career (combining artistic and non-artistic work) is the expected way to pursue a career in my professional environment.	3	57,38	3	50,77	*U* = 1258,00; *p* = 0,260
During my art education, I was prepared to have to change careers.	2	57,80	2	50,21	*U* = 1232,00; *p* = 0,194
During my art education, I was encouraged to improve my non-artistic competences.	3	57,89	3	50,11	*U* = 1227,00; *p* = 0,187
A dual career hinders the artistic progress of the professional performing artist *(inverted scale)*.	3	53,34	3	56,00	*U* = 1363,00; *p* = 0,653
During art education, one can prepare for other professions at the same time without much trouble.	3	51,58	3	58,29	*U* = 1255,50; *p* = 0,253

Source: own research.

**Table 12:** Degree of agreement with statements regarding the feasibility of dual career strategies, compared by career pursued (N = 111).

	Dual (n = 72)		Single (n = 39)		
Dependent variable	*Mdn*	*M*_rank_	*Mdn*	*M*_rank_	Mann-Whitney *U*
**A dual career (combining artistic and non-artistic work) is the expected way to pursue a career in my professional environment.**	**3**	**60,95**	**3**	**46,86**	***U* = 1047,50; *p* = 0,023**
During my art education, I was prepared to have to change careers.	2	57,10	2	53,96	*U* = 1324,50; *p* = 0,610
During my art education, I was encouraged to improve my non-artistic competences.	3	58,67	3	51,08	*U* = 1212,00; *p* = 0,220
A dual career hinders the artistic progress of the professional performing artist *(inverted scale)*.	3	59,40	2	49,72	*U* = 1159,00; *p* = 0,120
**During art education, one can prepare for other professions at the same time without much trouble.**	**3**	**60,40**	**3**	**47,88**	***U* = 1087,50; *p* = 0,043**

Source: own research.

In the next step of the analyses, it was investigated whether the individual statements were related to job tenure (Table 13). As part of the correlation analyses, a negative correlation of moderate strength (0.3 < rho < 0.5) was also found between the statement “During my art education, I was encouraged to improve my non-artistic competences” and general job tenure, *rho* = 0.31, *p*< 0.001, and between the above statement and artistic job tenure, *rho* = 0.40, *p*<0.001. A negative correlation of weak strength was also found between the statement “During art education, one can prepare for other professions at the same time without much trouble.”, *rho* = 0.22, *p* = 0.018.

**Table 13:** Correlations of survey items on potential career guidance strategies and job tenure.

		Job tenure (total)	Job tenure in the arts
A dual career (combining artistic and non-artistic work) is the expected way to pursue a career in my professional environment.	Spearman’s *rho*	-0,13	-0,13
	relevance	0,186	0,167
During my art education, I was prepared to have to change careers.	Spearman’s *rho*	-0,07	-0,16
	relevance	0,446	0,096
During my art education, I was encouraged to improve my non-artistic competences.	Spearman’s *rho*	-,314**	-,398**
	relevance	**<0,001**	**<0,001**
A dual career hinders the artistic progress of the professional performing artist *(inverted scale)*.	Spearman’s *rho*	-0,08	-0,10
	relevance	0,385	0,294
During art education, one can prepare for other professions at the same time without much trouble.	Spearman’s *rho*	-0,13	-,223*
	relevance	0,174	**0,018**

Source: own research.

## Discussion

Prioritising artistic careers over other professional or educational career paths is just one strategy for performing artists to pursue. Through an analysis of the body of subject literature, we have determined that diversification of career paths may be beneficial for a number of reasons for artists experiencing a lack of stability and predictability in the job market (in Poland and elsewhere).

Notably, a significant group of respondents in our survey said that it was the pandemic that made them aware of the instability of a career as a performing artist (56.7%). However, as many as 46.8% of respondents did not confirm that it was the pandemic that made them think about getting an education that would prepare them for a profession outside the arts. However, there were no statistically significant differences according to gender, age of general and artistic education, job tenure (total and artistic), profession pursued and dual career pursuit.

One may speculate that performing artists are aware of their personal limitations when it comes to the long-term pursuit of the artistic profession, but the pandemic has clearly exposed this group of creative workers to the importance of turbulent changes in the environment of cultural organisations. Our research showed that a significant group of respondents found the pandemic to be a driver for becoming aware of the fragility of an artistic career (56.7%), acknowledging that an artistic career does not provide a decent standard of living (43.2%), admitting the need to seek an alternative career path (47.7%), recognising the importance of undertaking an education that prepares one for a non-artistic profession (33.3%), and initiating the intention to seek additional professional work (44.1%).

In response to the second research question, we sought to find out artists’ attitudes towards artistic career end/descent. Interestingly, exactly 44 of the respondents stated that the decline of an artistic career is a failure in one’s professional life and exactly 44 denied this statement.

The study found some significant inter-gender differences in the analysed construct. It was found that men were more likely to agree with the statement “I will be able to give up my artistic career if it negatively affects my physical health”. Men were also more likely to declare that “I will be able to give up my artistic career if it does not provide me with a decent standard of living”. It seems that these differences are due to the family model that is still dominant in Poland, in which the man is supposed to be responsible for the family’s material standard of living to a greater extent than the woman. Thus, the issue of working for a decent wage and preserving the physical health needed for work may be a determinant for men to give up artistic work. One might suppose that these differences confirm that there is a rather conventional pattern of masculinity in Poland. On the other hand, the identified gender differences may be due to the fact that men in Poland still earn more than women, thus male respondents may have perceived more favourable non-artistic job offers in their environment. Therefore, artistic work does not necessarily entail such a high sacrifice, e.g. deterioration or loss of physical health, for them.

As part of the analyses, a positive correlation of weak strength was also found between total job tenure and the statement “I will be able to give up my artistic career if it interferes with my private life”. This result may suggest that as respondents gain work experience, they recognise the value of what happens in their non-work life. However, the observed relationship is so weak that it is hard to draw any firm interpretations.

The most surprising findings were yielded by the analysis of respondents’ views on alternatives to practising as a performing artist. As part of the analyses, it was found that men were more appreciative of artists’ support for retraining. This may be a matter of social support for men who want to gain additional professional qualifications. Institutional support, e.g. provided by institutions offering retraining programmes for artists, does not harbour signs of gender discrimination. Claims in this respect have not been made in the reports published so far on the job position of artists in Poland. Differences were also reported in the assessment of respondents according to the careers pursued. Those pursuing dual careers were more likely to agree with the statement indicating that “Pedagogical education makes it easier for artists to find a rewarding job after their artistic career is over” than those pursuing single careers. Parallel pedagogical work is a widely community-accepted way of existing in the artists’ job market. In many cases, achievements in teaching work provide the artist with a confirmation of their artistic mastery and craftsmanship, as well as a professional status confirmed by attracting talented students.

Very interesting results were obtained with the statement “Having only an art degree is sufficient for an artist to find a rewarding job after their artistic career is over”. Men and artists pursuing a single career are more in agreement with the quoted statement. The quoted statement correlates positively and weakly with total job tenure. Perhaps the respondents perceive values resulting from practising an artistic profession that can be useful in other than artistic work. This seems to be a point worth pursuing in research to find out what job satisfaction is, and which professions are sources of this satisfaction for artists who do not have a non-artistic background.

The purpose of our study was also to find out artists’ views on dual careers as sustainable careers. The evaluation of dual careers as an expected way of pursuing a career in a professional environment was rated better by those who were just pursuing dual careers. Those pursuing dual careers were also significantly more likely to agree with the statement “During art education, one can prepare for other professions at the same time without much trouble”. Thus, there are artistic environments that favour such a career model and allow it to come to fruition. In the meantime, it is worth continuing with the question of people who pursue single careers and exploring the characteristics of their backgrounds. In this case, do we talk about outstanding artists who have already established their professional status, about recognisable artists, for whom fame also brings material benefits, or about yet other situations specific to the artists’ job market?

As part of our correlation analyses, we also found a negative correlation of moderate strength between the statement “During my art education, I was encouraged to improve my non-artistic competences” and general job tenure and between the above statement and artistic job tenure. We also observed a negative correlation of weak strength between the statement “During artistic education, one can prepare for other professions at the same time without much trouble”. The present results bring the hope that people with shorter job tenure, perhaps just starting their artistic career, have just such expectations in order to secure their future and to arrange their career sequences in a conscious and balanced way. This is probably due to how art education in Poland is changing towards versatility, flexibility and the formation of interpersonal competences such as teamwork among students. New generations of artists are likely to contribute to a fresh perspective of cultural organisations on the management of artistic careers.

## Limitations

This study has several limitations that can guide future research. The results of our study, due to insufficient sampling, may not be generalised to the entire population of performing artists in Poland. We attempted to reach as many people meeting the sampling criteria as possible; however, we were only able to obtain fully completed online surveys from 111 people. The matched sample does not reflect the structure of the professional group of artists in Poland by age, gender or artistic profession.

Our study is also limited by the design of the tool itself. The use of only closed questions in the survey poses the danger of suggesting answers to the respondent, particularly when the respondent does not have a well-defined opinion on the issue addressed in the question. In addition, the theme of the survey may have encouraged respondents to “complain” about their professional status, which is portrayed in Polish media discourse as difficult due to the lack of sufficient social security for artists. The necessity to respond to the statements given in the survey by indicating the answers on a 5-point scale also presents somewhat of a drawback. It seems that difficult research topics, such as artists’ professional fates, decisions related to the continuation of their artistic careers or the need to assess their material position, require in-depth research, preferably conducted in an atmosphere of mutual trust with the researcher, intimacy, honesty and insight.

Unfortunately, our study was a one-off, which implies that it does not allow us to witness the variability of the phenomenon studied over time. It would be reasonable to replicate this study on the grounds that it was conducted at a time when artists were directly experiencing the consequences of social isolation caused by the pandemic. Currently, respondents may have different reflections and thoughts resulting from getting used to the new professional reality, as well as the new organisational solutions that cultural institutions have implemented.

## Conclusions

Based on the findings presented in this study, several key conclusions can be drawn regarding artistic careers and the attitudes of artists towards various professional pathways. Firstly, the COVID-19 pandemic has illuminated the inherent fragility of a career in the performing arts, prompting a notable number of artists to consider alternative employment options. While a significant proportion of respondents acknowledged the necessity for educational pathways that equip them for non-artistic professions, a substantial portion did not express readiness to pursue such alternatives. Furthermore, the analysis revealed gender-specific differences in the perception of artistic careers as successes or failures. Male respondents demonstrated a greater propensity to abandon their artistic careers should they adversely affect their physical health or overall standard of living. Additionally, those engaged in dual career trajectories exhibited a heightened appreciation for the value of pedagogical education, thereby underscoring the importance of institutional support for retraining initiatives. The evolving landscape of artistic education, which increasingly emphasizes the acquisition of non-artistic competencies, suggests a potential benefit for emerging generations of artists. Consequently, the future sustainability of artistic careers may hinge upon increased flexibility and adaptability, facilitating more effective career management within a dynamic labor market. These findings highlight the necessity for continued research into the attitudes and expectations of artists, thereby enhancing the understanding of their needs and opportunities within the professional realm of the arts.

## Supporting information

S1 Dataset (XLSX)

S1 File (DOCX)
