# Supplementary material for: Dual careers as sustainable careers for performing artists in times of crisis. A contextual approach to the construct of a sustainable arts career
Source: PLoS One. 2024 Dec 5;19(12):e0314933. doi: 10.1371/journal.pone.0314933 (PMC11620584; doi:10.1371/journal.pone.0314933)
Supplement: S1 File — (DOCX) [file pone.0314933.s002.docx]

Schedule No. 1. Research Tools

**SURVEY FORM**

Dear Artists,

This survey is intended only for people who are currently working in a performing arts profession (e.g. actress/actor, dancer, singer).

We are interested in hearing your views on the professional position of artists and potential opportunities to pursue dual careers. Our goal is to make recommendations for creating support programmes for artists that reflect the actual issues they face when managing their own careers.

Please fill in the survey below honestly. We guarantee complete anonymity.

**Thank you for participating in the survey,**

**Research Team**

**Rating scale:**

| **5** | **4** | **3** | **2** | **1** |
| --- | --- | --- | --- | --- |
| **I strongly agree** | **I rather agree** | **Hard to say** | **I rather disagree** | **I strongly disagree** |

**Please respond to the following statements:**

| **Item** | **Statement:** | **5** | **4** | **3** | **2** | **1** |
| --- | --- | --- | --- | --- | --- | --- |
| 1 | It was the pandemic that made me realise that a career as a performing artist is unstable. |  |  |  |  |  |
| 2 | It was the pandemic that made me realise that a career as a performing artist did not provide me with a decent standard of living. |  |  |  |  |  |
| 3 | It was the pandemic that made me realise that I should plan an alternative career path. |  |  |  |  |  |
| 4 | It was the pandemic that prompted me to pursue an education preparing me for a profession other than the performing arts profession. |  |  |  |  |  |
| 5 | It was the pandemic that prompted me to seek additional work in a profession other than the performing arts profession. |  |  |  |  |  |

**Please respond to the following statements:**

| **Item** | **Statement:** | **5** | **4** | **3** | **2** | **1** |
| --- | --- | --- | --- | --- | --- | --- |
| 6 | Ending an artistic career is a setback in an artist’s professional life. |  |  |  |  |  |
| 7 | I will be able to give up my artistic career if it negatively affects my physical health. |  |  |  |  |  |
| 8 | I will be able to give up my artistic career if it negatively affects my mental health (e.g. experiencing negative emotions, excessive workload, feeling stressed and pressured). |  |  |  |  |  |
| 9 | I will be able to give up my artistic career if it interferes with my private life (e.g. family, social life). |  |  |  |  |  |
| 10 | I will be able to give up my artistic career if it does not provide me with a decent standard of living. |  |  |  |  |  |

**Please respond to the following statements:**

| **Item** | **Statement:** | **5** | **4** | **3** | **2** | **1** |
| --- | --- | --- | --- | --- | --- | --- |
| 11 | Pedagogical education makes it easier for artists to find a rewarding job after their artistic career is over. |  |  |  |  |  |
| 12 | Non-artistic (other than pedagogical) education makes it easier for artists to find a rewarding job after their artistic career is over. |  |  |  |  |  |
| 13 | Having only an art degree is sufficient for an artist to find a rewarding job after their artistic career is over. |  |  |  |  |  |
| 14 | Formal support to artists for vocational retraining is properly implemented (e.g. through existing vocational retraining programmes). |  |  |  |  |  |
| 15 | Cultural institutions where performing artists are employed properly prepare artists for the need for professional reorientation (retraining). |  |  |  |  |  |

**Please respond to the following statements:**

| **Item** | **Statement:** | **5** | **4** | **3** | **2** | **1** |
| --- | --- | --- | --- | --- | --- | --- |
| 16 | A dual career (combining artistic and non-artistic work) is the expected way to pursue a career in my professional environment. |  |  |  |  |  |
| 17 | During my art education, I was prepared to have to change careers. |  |  |  |  |  |
| 18 | During my art education, I was encouraged to improve my non-artistic competences. |  |  |  |  |  |
| 19 | A dual career hinders the artistic progress of the professional performing artist. |  |  |  |  |  |
| 20 | During art education, one can prepare for other professions at the same time without much trouble. |  |  |  |  |  |

**Please respond to the following statements:**

| **Item** | **Statement:** | **5** | **4** | **3** | **2** | **1** |
| --- | --- | --- | --- | --- | --- | --- |
| 21 | I have a high level of mental resilience. |  |  |  |  |  |
| 22 | I cope with work stress well. |  |  |  |  |  |
| 23 | I adapt easily to new working conditions. |  |  |  |  |  |
| 24 | I learn new things quickly. |  |  |  |  |  |
| 25 | I can work together as part of a team. |  |  |  |  |  |

**Metrics**

**Gender:** female-0 male-1 other gender-2 I don’t want to answer-3

**Age:** up to 20 years-1; 21-30 years-2; 31-40 years-3; 41-50 years-4; 51-60 years-5;

**General education:**

Primary-1, lower secondary-2, vocational-3, secondary-4, tertiary-5

**Artistic education:**

None-1, graduated from primary art school-2
graduated from secondary art school-3, graduated from tertiary art school-4

**Job tenure (total):**

Less than one year 1-5 years-1; 6-10 years-2; 11-15 years-3;
16-20 years-4; over 20 years-5

**Job tenure as an artist:**

Less than one year 1-5 years-1; 6-10 years-2; 11-15 years-3;
16-20 years-4; over 20 years-5

**Performing arts occupation**: singer; actor/actress; dancer; instrumentalist, other, if yes, please specify

Please specify

**Are you pursuing a dual career, i.e. working simultaneously as a performing artist and in another profession (e.g. as an educator)?**

Yes-1 No-2

**If yes, what profession (other than arts) do you pursue?**

Please specify
